# Critical Analysis of the Commercial Potential of Plants for the Production of Recombinant Proteins

**DOI:** 10.3389/fpls.2019.00720

**Published:** 2019-06-11

**Authors:** Stefan Schillberg, Nicole Raven, Holger Spiegel, Stefan Rasche, Matthias Buntru

**Affiliations:** ^1^ Fraunhofer Institute for Molecular Biology and Applied Ecology IME, Aachen, Germany; ^2^ Institute for Phytopathology, Justus-Liebig-University Giessen, Giessen, Germany; ^3^ Aachen-Maastricht Institute for Biobased Materials, Geleen, Netherlands

**Keywords:** cell-free biosynthesis, CHO cells, molecular farming, plant-made pharmaceuticals, *Pseudomonas fluorescens*

## Abstract

Over the last three decades, the expression of recombinant proteins in plants and plant cells has been promoted as an alternative cost-effective production platform. However, the market is still dominated by prokaryotic and mammalian expression systems, the former offering high production capacity at a low cost, and the latter favored for the production of complex biopharmaceutical products. Although plant systems are now gaining widespread acceptance as a platform for the larger-scale production of recombinant proteins, there is still resistance to commercial uptake. This partly reflects the relatively low yields achieved in plants, as well as inconsistent product quality and difficulties with larger-scale downstream processing. Furthermore, there are only a few cases in which plants have demonstrated economic advantages compared to established and approved commercial processes, so industry is reluctant to switch to plant-based production. Nevertheless, some plant-derived proteins for research or cosmetic/pharmaceutical applications have reached the market, showing that plants can excel as a competitive production platform in some niche areas. Here, we discuss the strengths of plant expression systems for specific applications, but mainly address the bottlenecks that must be overcome before plants can compete with conventional systems, enabling the future commercial utilization of plants for the production of valuable proteins.

## Introduction

The function of a protein is determined by the number and sequence of amino acids, which controls the three-dimensional structure of the resulting folded polypeptide. Proteins are therefore molecules of great complexity and near infinite diversity, making them suitable for many different applications. More than 300 protein-based medicines have been approved in the USA and Europe, and proteins account for almost a third of all pharmaceuticals in development (Walsh, 2018). Proteins are also widely used in industry, including enzymes used to manufacture textiles and chemicals and to process food and feed. Many other proteins are used as diagnostics or research reagents. The demand for recombinant proteins is therefore rising steadily, with a market valued at US$1.654 billion in 2017 predicted to reach US$2.850 billion by 2022 (Markets and Markets, 2017). Therapeutic proteins (e.g., antibodies, vaccines, enzymes, cytokines, and growth factors) account for almost half of this market, followed by industrial proteins (e.g., technical enzymes) and research reagents (e.g., antibodies for protein detection and purification) (Markets and Markets, 2017). Market growth has been supported by advances in recombinant protein production technologies, including the engineering of expression hosts, the optimization of upstream cultivation (e.g., bioreactor design, nutritional, and physical parameters), and the development of more efficient protein extraction and purification methods. Most recombinant proteins are currently produced in prokaryotic cells (mainly the bacterium *Escherichia coli*) and a small number of well-characterized mammalian cell lines, such as Chinese hamster ovary (CHO) cells. Other systems are used in commercial processes but are less common, including insect cells, yeast, algae, and cell-free expression platforms (Markets and Markets, 2017). There are also several platforms based on plants and plant cells, but these have not been included in the latest market studies, indicating they have not yet commanded a significant share of commercial protein production capacity. Even so, plants as an alternative expression platform offer unique advantages, particularly when target proteins are difficult to produce in conventional systems, require specific qualitative properties such as particular glycan profiles, and/or must be produced on a larger scale in response to urgent demand. Improvements in expression levels and the economics of downstream processing will promote the utilization of plants for commercial protein production.

### Conventional Expression Systems – Mammalian and Prokaryotic Cells

Industry favors recombinant protein expression systems that have a long and successful track record, specifically with three goals in mind: high quality, high yields, and low costs. In addition, such systems should meet the demands of an industrial process with respect to robustness, and economic sustainability, and must comply with regulatory requirements. This is particularly relevant for pharmaceutical proteins produced according to good manufacturing practice (GMP), a set of guidelines ensuring that biopharmaceuticals are sufficient in terms of quality and batch-to-batch consistency in order to prevent harm to patients. The industry has therefore focused its resources on a small number of cell-based systems, in particular CHO cells and *E. coli*, which are now considered the gold standards for industrial protein manufacturing.

Many complex proteins, including most therapeutic antibodies, are routinely produced in CHO cells because they have the capacity to carry out authentic post-translational modifications, including glycosylation. Recombinant proteins produced in CHO cells are secreted into the culture medium to facilitate recovery and purification. Various strategies have been pursued to maximize the productivity of CHO cell cultures, including: (1) the engineering of production lines and expression vectors, (2) amplification of the expression cassette, (3) optimization of the cell culture medium, including the switch from early formulations containing serum to chemically defined and near protein-free formulations that simplify protein purification even further, (4) increasing the cell density during cultivation, and (5) the introduction of fermentation strategies that balance the nutrient supply while maintaining optimal cultivation conditions (Hausmann et al., 2018; Ritacco et al., 2018). These developments have led to remarkable increases in yields. For example, early processes for the manufacture of monoclonal antibodies achieved titers of hundreds of milligrams per liter, but this has increased to routine titers of 5–10 g/L and in some cases up to 20 g/L (Pujar et al., 2017), reducing the cost of goods to as little as €20/g (Kelley, 2007). Combined with GMP-compliant cell lines and processes and well-established approval procedures, it is hard to imagine that the CHO platform will be displaced by any other expression system for the manufacturing of complex proteins in the near future.

**Figure 1:**
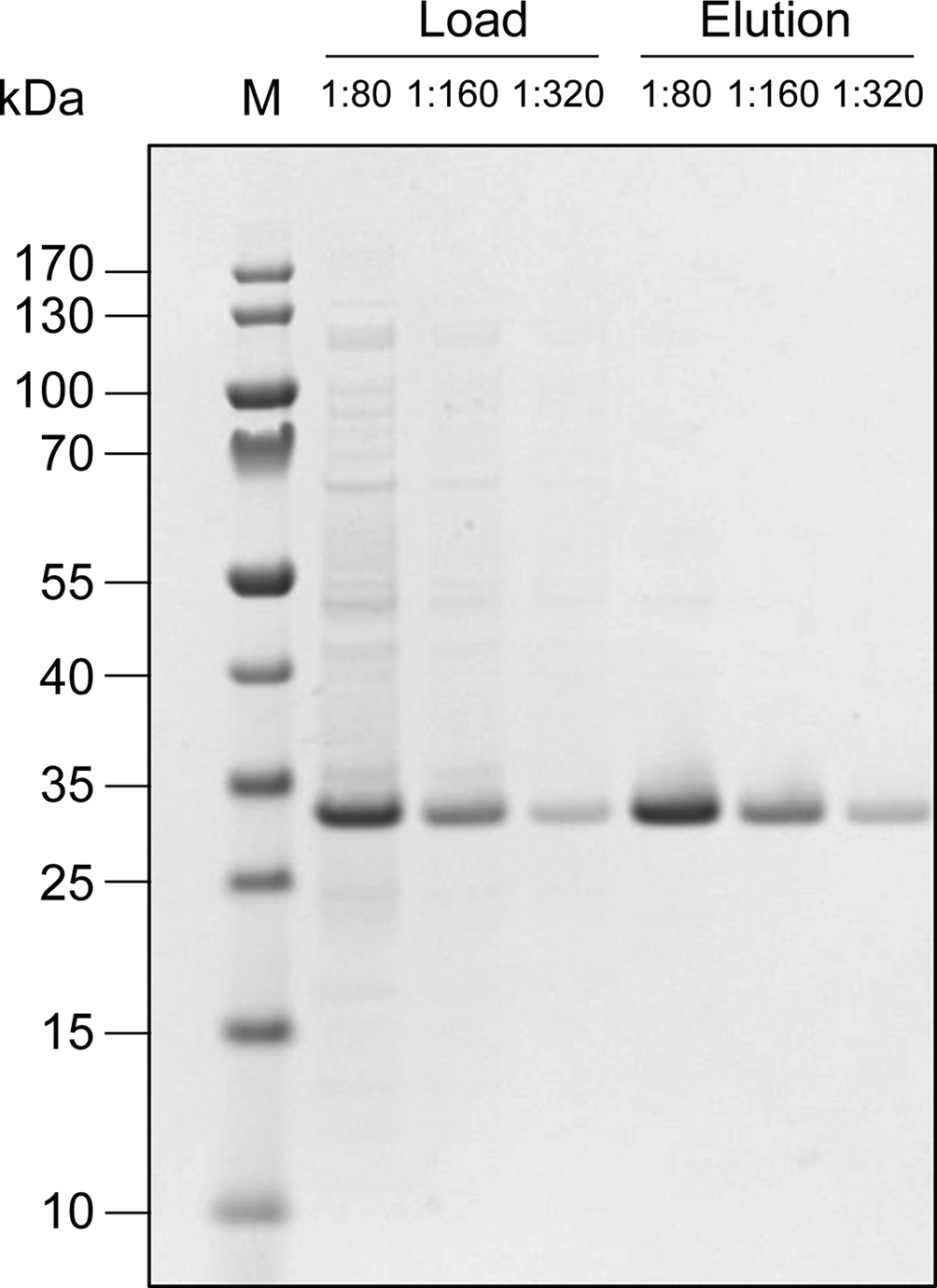
Production of a 19-kDa phenylalanine-free protein in *P. fluorescens*. After extraction and the removal of cell debris, the product was purified from the clarified extract by single-stage immobilized metal-ion affinity chromatography. Due to the high protein concentration, representative samples of the load and elution fraction were analyzed by SDS-PAGE at dilution ratios of 1:80, 1:160, and 1:320. The strong band at ~32 kDa represents the phenylalanine-free protein – the larger size of the protein probably reflects a combination of its high surface charge and generally high stability, which prevents full de-folding during sample preparation. This example shows the high efficiency of the purification step: only traces of other proteins are found in addition to the target protein in the final elution fraction, corresponding to a purity of >95%.

Although mammalian cells are favored for the production of complex proteins, prokaryotic cells are much easier to handle and are much less expensive in terms of media requirements. Accordingly, where the product is a simpler protein, *E. coli* is often the ideal choice of production host. Indeed, the first recombinant therapeutic protein (human insulin) has been commercially produced in *E. coli* since 1982 (Baeshen et al., 2014). Many other commercial recombinant protein products including cytokines for cancer treatment or technical enzymes for industrial applications have been produced in *E. coli*, but its status as the gold standard prokaryotic host is mainly for historical reasons and a range of other prokaryotes may be more suitable (Sanchez-Garcia et al., 2016; Singh et al., 2016). In our laboratory, we use *Pseudomonas fluorescens* for the larger-scale production of recombinant proteins, which accumulate in the cytosol by default or can be secreted to the culture medium (Retallack et al., 2012). For example, we chose cytosolic accumulation for the production of a 19-kDa phenylalanine-free protein that can be used for the dietetic management of patients suffering from phenylketonuria – an inborn error metabolism that results in decreased metabolism of the amino acid phenylalanine (Hoffmann et al., 2018). The phenylalanine-free protein can easily be extracted from the cells by high-pressure homogenization and isolated *via* a single affinity purification step (Figure 1). Simple medium-scale cultivation in 2.5-L shake flasks with a culture volume of 0.5–1.0 L achieved yields of 2.5 g/L. Fed-batch fermentation in bioreactors with a working volume of 5–350 L increased productivity to 20 g/L, enabling the production of 3.5 kg of the target protein for animal tests within a few weeks and demonstrating the feasibility of a scaled-up industrial process that provides ton quantities as a supplement for phenylalanine-free food production.

Despite the availability of high-performance protein expression hosts, there is a constant demand for improved or completely new systems to reduce manufacturing costs by increasing productivity, quality, and/or yields. During the late 1980s and early 1990s, plants and plant suspension cell cultures were proposed as alternative production systems (Hiatt et al., 1989; Fischer et al., 1999). In particular, the scalability of plant-based systems combined with the low cost of plant cultivation was predicted as a major driver to reduce manufacturing costs. However, this promise has yet to be fully realized, mainly reflecting the low yields of plants and the high costs of product recovery and purification.

### Plant-Based Production Systems and Plant-Derived Protein Products

Since the 1990s, many researchers have aspired to produce recombinant proteins in plants. Typically, they favored plants that were already used for other research purposes because the techniques required for gene transfer were readily available. This led to the development of an extremely diverse array of production systems, including whole plants, various tissue and cell systems (hairy roots and cell suspension cultures), and numerous expression approaches (stably transformed transgenic and transplastomic plants, transient expression systems, inducible expression, and different protein targeting strategies; Twyman et al., 2003, 2005; Schillberg et al., 2013; Spiegel et al., 2018). A suitable platform is therefore likely to be available for any conceivable product, but the absence of a standard platform scatters and slows down efforts to optimize productivity and makes it more difficult to define industrial production standards.

In terms of product candidates, research has focused mainly on biopharmaceuticals with a higher added-value compared to diagnostic and technical proteins. In this context, three main protein product classes have emerged: antibodies, vaccine candidates, and replacement human proteins such as blood products (human serum albumin), replacement proteins for common and rare diseases (gastric lipase for cystic fibrosis, insulin for diabetes, glucocerebrosidase for Gaucher’s disease), or growth factors and cytokines (Spiegel et al., 2018). Recombinant antibodies, antibody fragments, and antibody fusion proteins have become the most common products expressed in plants (Nölke et al., 2003; Vasilev et al., 2016) because they are both economically important as pharmaceuticals (Walsh, 2018) and also relatively stable and easy to characterize. This means they accumulate to high levels (>100 mg/kg fresh plant weight or > 100 mg/L culture medium), are easy to purify even from complex plant matrices, and their functionality can be verified using simple binding assays. However, antibody titers in plants still lag far behind the yields currently achieved in CHO cells, making it uncertain that plants will ever become suitable as a routine commercial platform for antibody products. As discussed below, however, there are certain niche markets where plants offer capabilities that cannot be matched by CHO cells or any other platform.

Many studies involving the production of recombinant proteins in plants have not ventured into the hard realities of commercial development and have focused instead on early-stage objectives such as verifying expression, optimizing production and purification to a certain extent, and the completion of initial functionality assays. Few studies have included translational research demonstrating commercial competitiveness, partly due to the financial and organizational challenges that must be overcome before plant-derived biopharmaceuticals can be tested in clinical trials. It is almost impossible to secure financial and business support if the market potential and intellectual property rights are unclear, as is the case for most protein products made in plants. But without a solid business case industry will not switch from its established microbial and mammalian production systems to plants because the risk would not be justified. Nevertheless, a few plant-derived biopharmaceutical product candidates have entered clinical trials, helping to define GMP-compliant processes now approved by the regulatory authorities (Fischer et al., 2012, 2013; Sack et al., 2015). A handful have reached the market, the first of which was recombinant glucocerebrosidase (prGCD), generic name taliglucerase alfa, marketed as Elelyso, which is manufactured in carrot cells by Protalix Biotherapeutics (Rup et al., 2017; Zimran et al., 2018).

Given the long timelines and huge investment needed to provide proof of concept for the business potential of plant-derived biopharmaceuticals, it may be better to pick the low-hanging fruit. This means products that allow quicker access to the market due to the less-stringent regulatory requirements, as is the case for diagnostic, technical, and cosmetic products. Key examples include the diagnostic reagent avidin, which was first commercially produced in maize 20 years ago (Hood et al., 1997) and is still sold by Sigma-Aldrich (catalog no. A8706) and human epidermal growth factor produced in barley as a cosmetics additive, distributed by Sif Cosmetics (Iceland). However, two major challenges that must be addressed before plants can become more generally competitive with other expression systems are the low product yields and the cost of downstream processing.

## Challenges Facing Recombinant Protein Production in Plants

In 1995, a secretory antibody was produced in tobacco plants with a titer of 500 μg/g fresh plant material (Ma et al., 1995). Although this has been exceeded by model proteins such as green fluorescent protein, which was transiently expressed in *Nicotiana benthamiana* leaves with a yield of 4 mg/g fresh weight (Marillonnet et al., 2005; Yamamoto et al., 2018), or the *Bacillus thuringiensis* (*Bt*) Cry2Aa2 protein, which accumulated as crystals in tobacco chloroplasts with a yield of ~5 mg/g fresh weight (De Cosa et al., 2001), other products including antibodies rarely accumulate to levels exceeding 100 μg/g fresh weight. This is despite extensive research to optimize protein expression and stability in plants by addressing internal factors (e.g., expression cassettes, protein targeting strategies, and the co-expression of protease inhibitors) and external factors (e.g., nutritional and physical cultivation parameters affecting plant growth and fitness) (Twyman et al., 2013). In addition, the challenge of protein purification from complex plant matrices reduces final yields while contributing to the high overall manufacturing costs.

### The Yield Challenge

Recombinant protein yield is defined by the intrinsic productivity of the host, the biomass of the expression host in a given volume or area, and the potential for scale-up. Most approaches for yield improvement aim to increase the cell-specific productivity (qP) by genetic engineering or optimizing the culture conditions. In optimized CHO bioprocesses, the qP can reach 50–90 pg per cell per day, whereas human secretory plasma cells are capable of secreting IgM at a rate of 200–400 pg per cell per day (Hansen et al., 2017). It is rare to find qP values quoted for plant-based systems. For a tobacco cell suspension culture with a maximum yield of 100 mg/L for a full-size antibody, the equivalent qP value is 8.0 pg per cell per day (Havenith et al., 2014). Although this is an order of magnitude lower than the maximum qP values of elite CHO cell lines, it is nevertheless promising because the further optimization of protein productivity in plants appears to be possible by controlling genetic and epigenetic factors, as well as cultivation parameters (Twyman et al., 2013).

A major limitation of plant cells is their size. Compared to plant cells, bacterial and mammalian cells are rather small and reach a packed cell volume (PCV) of less than 5% in conventional batch cultures. Therefore, a common strategy to increase productivity is to increase the cell density/number in industrial production process leading to PCVs of almost 50%. In contrast, plant cells have a significantly larger volume caused mainly by the presence of dominant vacuolar compartment. The large size of plant cells means that productivity cannot be enhanced in suspension cultures by increasing the cell number, because at the end of the cultivation period, the PCV is already 60–80% of the culture volume. Plant cell size can be reduced by increasing the osmolality of the culture medium to shrink the vacuole, resulting in higher cell numbers at the same PCV, but even then plant cells remain much larger than both mammalian cells and bacteria (Vasilev et al., 2013).

Protein yield can also be enhanced by increasing the production volume. This involves a simple process of scaling up when dealing with plant cell suspension cultures, but the costs also increase, thus not helping to improve commercial feasibility (Raven et al., 2015). The situation is different for whole plants, which have the capacity to produce much more biomass than conventional fermenters and at lower costs, even when cultivation is restricted to greenhouses. Even so, increasing yields by boosting overall biomass production rather than intrinsic productivity transfers demand to the protein extraction and purification steps, and this must be considered with regard to the overall production costs as described in “The Purification Challenge.”

Ultimately, plants offer a high capacity for protein synthesis but the production unit, represented by a single cell, is too large to be competitive with the smaller cells of conventional protein production systems, which therefore achieve greater productivity on a smaller footprint. One solution is to remove all the unnecessary components from plant cells, e.g., the vacuole, to concentrate their production capacity and simultaneously eliminate factors that decrease protein yields, such as endogenous plant proteases (Schiermeyer et al., 2005; Mandal et al., 2016). In a step toward this goal, the plant protein synthesis machinery has been separated from unnecessary and undesirable components by preparing plant cell-free lysates. The most widely used lysates are prepared from wheat germ embryos and contain everything necessary for transcription and translation, but extensive washing during extract preparation removes translational inhibitors so that the resulting *in vitro* transcription-translation reactions achieve yields of 100 μg/ml in a single batch process (Biotechrabbit, 2019a). In a dialysis bag continuously fed with substrates and with small inhibitory byproducts continually removed, the yields can reach 1,000 μg/ml (Biotechrabbit, 2019b). However, the preparation of wheat germ extracts is time consuming and expensive, and the potential for scale-up is limited to the milliliter range (Takai et al., 2010).

**Figure 2:**
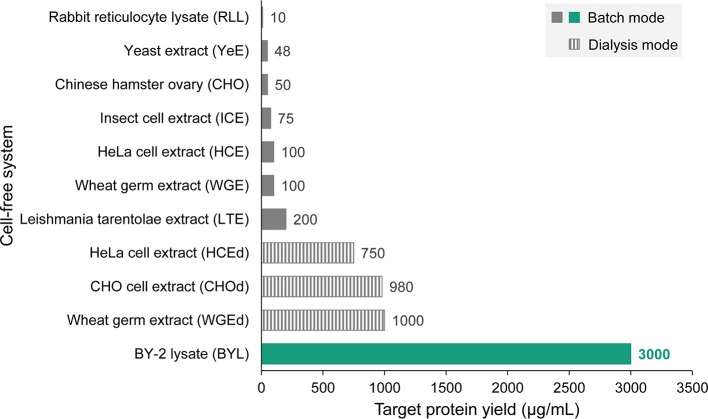
Comparison of different eukaryotic cell-free expression systems with the tobacco BY-2 cell-free lysate (BYL). Cell-free reaction modes (coupled batch or continuous feeding of substrates and removal of inhibitory byproducts by dialysis) are indicated. The data represent the highest protein yields reported for each system including the information which target protein has been used to determine maximum product levels and are sourced from the following references and company information: rabbit reticulocyte lysate (RLL, luciferase) (promega.de), *Pichia pastoris* yeast extract (YeE, human serum albumin) (Aw and Polizzi, 2018), CHO cell extract (luciferase) (Brodel et al., 2014), *Spodoptera frugiperda* insect cell extract (ICE, procaspase 3) (promega.de), HeLa cell extract (HCE, model protein not indicated) (thermofisher.com), wheat germ extract (WGE, model protein not indicated) (biotechrabbit.com), *Leishmania tarentolae* extract (LTE, eGFP) (jenabioscience.com), HCEd (model protein not indicated) (thermofisher.com), CHOd (epidermal growth factor-eYFP fusion), and WGEd (model protein not indicated) (biotechrabbit.com), and BYL (eYFP).

Recently, we described a new cell-free lysate based on tobacco BY-2 cells. The BY-2 lysate (BYL) achieves yields of up to 270 μg/ml when producing the fluorescent protein eYFP and involves a coupled transcription-translation reaction in a simple 18-h batch process (Buntru et al., 2014, 2015; Havenith et al., 2017). The productivity of the BYL system has been increased to 3,000 μg eYFP per ml by optimizing lysate preparation and the reaction components, and by extending the transcription-translation process to 24–48 h by including active mitochondria that deliver energy for protein biosynthesis (unpublished data). Although model proteins like eYFP are known to accumulate to very high levels, the maximum yields of the optimized BYL are 15-fold higher than any other eukaryotic batch-based cell-free system expressing similar proteins (Figure 2), demonstrating the enormous capacity of tobacco cell suspension cultures for protein biosynthesis. The BYL system has been commercialized by the company LenioBio and is marketed under the brand name ALiCE^1^. Lysate volumes of up to 150 ml can be prepared within a few hours by isolating protoplasts, followed by the removal of the vacuole (which contains most of the nucleases and proteases that reduce protein yields) by density centrifugation, and the final mechanical disruption of the evacuolated protoplasts (Buntru et al., 2015). Interestingly, the BYL system contains microsomes, vesicles generated by the disruption of the endoplasmic reticulum during lysate preparation. Therefore, proteins can be targeted to the microsomes by including N-terminal signal peptides, enabling the formation of disulfide bonds and the efficient folding and assembly of complex and multimeric functional proteins such as enzymes, full-size antibodies, and even membrane proteins. In addition, targeting to the microsomes enables *N*-linked protein glycosylation. However, elucidation of the detailed glycan pattern is still pending. Cell-free platforms are predominantly used at smaller scales (~50 μl) for screening and protein optimization, but the preparation and scale-up of the BYL is simple and inexpensive, and cell-free reactions have already been completed at the 6-ml scale. It therefore appears feasible that a further scale-up to 1–10 L will enable the production of gram quantities of recombinant proteins, especially those which are difficult to produce in cell-based systems due to their toxicity, instability or incompatibility with intracellular enzymes such as kinases (Huck et al., 2017).

### The Purification Challenge

**Figure 3:**
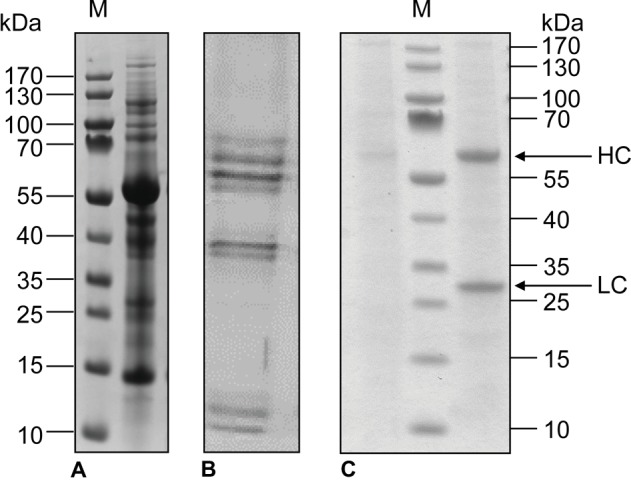
SDS-PAGE analysis of total soluble proteins in *N. benthamiana* leaf extracts **(A)**, tobacco BY-2 culture medium **(B)**, and CHO culture medium **(C)** without (left) and with (right) secreted monoclonal antibody (100 μg/ml). The positions of the antibody light chain (LC) and heavy chain (HC) are indicated. M: PageRuler Pre-stained Protein Marker.

Industrial processes for the extraction and purification of recombinant proteins produced by microbial and mammalian cells are well established, although downstream processing is still the main driver of overall costs (Singh and Herzer, 2018). Protein recovery is particularly straightforward when products are secreted to the culture medium by cells growing in suspension, which is generally the case for CHO cells. Therefore, the use of synthetic, protein-free medium for the cultivation of CHO cells avoids contaminating the product with endogenous host cell proteins almost completely (Figure 3C). In contrast, recombinant proteins produced in whole plants have to be extracted from the plant material, requiring the elimination of large quantities of insoluble debris and soluble plant host cell proteins during downstream processing (Figure 3A). Even when recombinant proteins are secreted by plant cell suspension cultures, the medium also contains several secreted host cell proteins that complicate product purification (Figure 3B).

**Figure 4:**
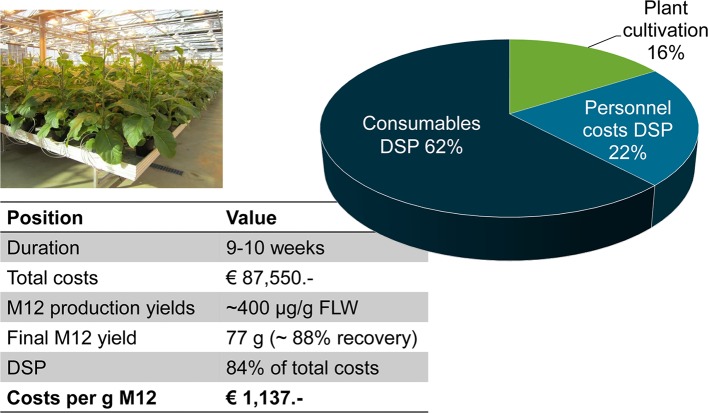
Costs for the production and purification of the human M12 antibody produced in transgenic tobacco plants in a contained greenhouse. The duration of the whole process from sowing to analysis of the purified protein was 10 weeks. We processed 200 kg of leaf material to provide 77 g of highly-purified M12 antibody. FLW, fresh leaf weight; DSP, downstream processing.

To determine the manufacturing costs for a plant-based process, we produced the human full-size antibody M12 (Kirchhoff et al., 2012) in tobacco plants and purified the recombinant protein from the plant matrix. We grew 1,440 transgenic, homozygous T_4_ *Nicotiana tabacum* cv. Petit Havana SR1 plants in the greenhouse, and the pre-purification antibody yield was 400 μg/g fresh leaf tissue (Figure 4). The presence of a KDEL signal peptide on the C-terminus of the heavy chain caused the recombinant antibody to accumulate in the endoplasmic reticulum. We harvested 200 kg of leaf material 8 weeks after sowing, and total soluble proteins were extracted using a custom-designed large-scale processing unit. To remove insoluble debris, the plant extract was passed over a sequential filtration cascade consisting of an initial bag filtration followed by three depth filtration steps with exclusion sizes of 8, 1, and 0.3 μm, respectively. Additionally, the clarified plant extract was passed through a 0.2-μm filter module before filling into disposable bags used for storage to avoid bacterial contamination. The M12 antibody was purified from the clarified plant extract using four process steps, i.e., Protein A chromatography, CaptoAdhere chromatography, ultrafiltration, and final diafiltration. The purity of the antibody in the final eluate was estimated to be >90%. The eluate from the final filtration step contained 0.5–5.0 units/ml of endotoxin and some residual Protein A (5–20 ng/mg IgG). The total yield of the purified product was 77 g, corresponding to a rather high recovery of 88% (Figure 4). The total process cost was €87,550 including labor, consumables, and infrastructure depreciation for plant cultivation and downstream processing, and all necessary analytics, which was equivalent to €1,137 per gram of purified antibody.

Plant-based systems are often described as cost-effective due to the low cost of upstream cultivation. However, as discussed for the M12 antibody above, cultivation accounts for only 16% of total process costs, whereas downstream processing represents the lion’s share of these costs due to the effort required to extract the protein from the intracellular environment and remove not only insoluble components of the plant matrix but also the many soluble host cell proteins that are released along with the product during homogenization. Recovering the same antibody from the culture medium of tobacco BY-2 cells involved much less effort, but downstream processing nevertheless accounted for 77% of total process costs (Raven et al., 2015). The productivity of the BY-2 cells was 20-fold lower than whole tobacco plants, thus the cost of goods for the purified antibody was 10-fold higher than the same product extracted from whole plants.

The low space yield and high cost of downstream processing are major weaknesses limiting the commercial utilization of plant-based production systems. In contrast, CHO cells achieve high antibody titers and the downstream process is straightforward, reducing the cost of goods to generally around US$200/g (Lim et al., 2010) and in exceptional cases to less than US$25/g (Kelley, 2007). As discussed above, many groups are pursuing various strategies to boost protein yields in plants, albeit with only limited success (Twyman et al., 2013). Another promising approach to make plant-based production more attractive is to reduce the costs of downstream processing, e.g., by heating plant extracts to achieve the rapid and efficient precipitation of host cell proteins (Beiss et al., 2015), or by developing new affinity ligands to improve purification efficiency (Ruhl et al., 2018). Plant-based production could also become more competitive by incorporating value from biomass side streams, especially for the recovery of bioactive plant proteins and small molecules, or by using the residual biomass to generate biogas (Buyel, 2019).

## Opportunities for Plant-Based Production

Although plants cannot compete with microbes or mammalian cells for most protein manufacturing processes, they become much more attractive in market niches relying on one or more of the following unique features:

▪ *Improved protein functionality*. Many biopharmaceuticals contain *N*-linked glycans, but plant glycans differ slightly from human glycans, especially the core *β*(1,2)-xylose and *α*(1,3)-fucose residues that are found in plants but not in endogenous mammalian glycoproteins. Therefore, several studies have focused on the humanization of glycan chains in plants by knocking out plant glycosyltransferases and introducing their human counterparts to avoid any adverse reactions when plant-derived biopharmaceuticals are injected into patients (Montero-Morales and Steinkellner, 2018). In contrast, vaccines and certain biopharmaceuticals for cancer immunotherapy may benefit from plant glycans because the immunogenicity stimulates the activity of antigen-presenting cells, particularly *via* lectins or mannose/fucose receptors on the surface of dendritic cells (Rosales-Mendoza et al., 2016). Furthermore, some therapeutic proteins carrying plant-derived glycans function in a superior manner to their native counterparts. One example is Elelyso, the recombinant form of human glucocerebrosidase mentioned above. This is produced in carrot cells and is targeted to the vacuole because the vacuole-specific glycans improve the uptake of the protein by human macrophages (Rup et al., 2017). Another example is the production of plant allergens requiring the proper presentation of plant glycans to enable the detection of IgE antibodies against plant cross-reactive carbohydrate determinants.
▪ *Plant matrix*. Plants offer an affordable strategy for the production of animal vaccines and therapeutics, especially if they can be administered topically or orally because this avoids the need for expensive downstream processing, reducing the manufacturing costs enough to make such veterinary products much more economically competitive. Oral vaccines and therapeutics can be administered directly as unprocessed or minimally plant tissues. The plant matrix may enhance the effectiveness of vaccines and therapeutics by encapsulating them and protecting them from digestion, thus extending the window of opportunity for exposure to the immune system or target cells. In one example, a poultry vaccine produced in tobacco suspension cells was injected as a crude extract into chickens to protect them against the Newcastle disease virus (Schillberg et al., 2013). In another example, peas expressing a parasite-specific antibody were administered to chickens to prevent gastrointestinal coccidiosis (Zimmermann et al., 2009).
▪ *Speed of production*. Transient expression systems allow the production of recombinant proteins within a few days. Therefore, this is particularly suitable for the production of emergency vaccines (e.g., influenza vaccines), which are needed within a few weeks or months after confirming the gene sequence. Transient expression is easy to scale up by carrying out the process of agroinfiltration in large vacuum tanks to generate gram quantities of the final product (Spiegel et al., 2015).
▪ *Consumer acceptance*. The production of recombinant proteins in plants might also improve the perception of the final product. This is particularly relevant in the case of cosmetic products because plants are recognized as more natural and environmentally sustainable than microbial and mammalian cells. Indeed, the production of human growth factor in barley plants (see above) is advertised with pictures of green plants growing in the greenhouse.
▪ *Animal-free production*. Plant systems allow the production of recombinant proteins without the use of any animal-derived reagents, which is required when producing therapeutics and vaccines for some religious communities, vegans or people with animal allergies. In addition, it is advantageous to produce diagnostic proteins without contaminating animal proteins or endotoxins to avoid interference in tests with mammalian cells, tissues or organs.

## Conclusions and Outlook

Despite their low productivity, large production footprint and high downstream processing costs compared to traditional platforms, plants, and plant cells possess some unique selling points which make them attractive for specific product lines such as proteins requiring plant-specific glycans, veterinary therapeutics, emergency vaccines, and animal-free proteins. However, the translation of many of these products from research to market is slow, limiting the visibility and commercial exploitation of plant-based platforms for recombinant protein production. Commercial translation is more straightforward and faster for non-therapeutic proteins because of the lower regulatory burden. These products are therefore more suitable in the first instance to demonstrate the economic sustainability of plant-based production systems. In contrast, therapeutic proteins promise higher profit margins but their commercialization requires comprehensive and expensive preclinical and clinical studies with backing from industrial partners providing expertise in drug development. Many studies concerning the production of therapeutic proteins in plants therefore never go beyond expression, purification, and cell-based analysis. Progress along the value chain requires additional work, including toxicity studies, the identification of biomarkers for patient stratification and therapeutic monitoring, clinical trials, and acceptance by the health insurance and regulatory agencies. Further commercial considerations include the intellectual property/freedom to operate portfolio, market share, potential competitors, and time to market. The launch of more plant-derived biopharmaceuticals on the market will require significant investment and closer cooperation with the pharmaceutical industry, regulatory authorities, and clinicians. Importantly, this only makes sense if the product offers unique advantages in terms of quality, efficacy, production scale/timing and/or cost when it is produced in plants rather than CHO cells or microbes.

## Data Availability

All datasets generated for this study are included in the manuscript and/or the supplementary files.

## Author Contributions

SS wrote the manuscript. NR, HS, SR, and MB revised the manuscript.

### Conflict of Interest Statement

SS is member of the Scientific Advisory Board of LenioBio GmbH distributing the BYL platform developed by Fraunhofer IME and Dow AgroSciences.

The remaining authors declare that the research was conducted in the absence of any commercial or financial relationships that could be construed as a potential conflict of interest.

## Footnotes

^1^www.leniobio.com/about-alice
